# Neurovascular alterations of muscularis propria in the human anterior vaginal wall in pelvic organ prolapse

**DOI:** 10.1111/joa.13014

**Published:** 2019-05-30

**Authors:** R Sferra, S Pompili, A D'Alfonso, G Sabetta, E Gaudio, G Carta, C Festuccia, A Colapietro, Antonella Vetuschi

**Affiliations:** ^1^ Department of Biotechnological and Applied Clinical Sciences University of L'Aquila L'Aquila Italy; ^2^ Department of Life, Health and Environmental Sciences, Gynecology and Obstetrics Unit University of L'Aquila L'Aquila Italy; ^3^ Department of Anatomical, Histological, Forensic Medicine and Orthopedic Sciences Sapienza University of Rome Rome Italy

**Keywords:** immunological staining, muscularis, neurovascular markers, pelvic organ prolapse

## Abstract

In the pathophysiology and progression of pelvic organ prolapse (POP), it has been demonstrated that there is a reorganisation of the muscularis propria of the anterior vaginal wall due to a phenotypic smooth muscle cell to myofibroblast switch. An abnormal deposition of collagen type III seems to be influenced by the involvement of advanced glycation end‐products. The aim of the present study was to evaluate the hypothesis that this connective tissue remodelling could also be associated with neurovascular alterations of the muscularis in women with POP compared with control patients. We examined 30 women with POP and 10 control patients treated for uterine fibromatosis. Immunohistochemical analysis, using glial fibrillary acidic protein, S‐100 protein, receptor tyrosine kinase, neurofilament and α‐smooth muscle actin antibodies, was performed. S‐100, receptor tyrosine kinase and neurofilament were also evaluated using Western blot analysis. We observed a decrease in all neurovascular‐tested markers in nerve bundles, ganglia and interstitial cells of Cajal from POP samples as compared with controls. Even if the processes responsible for these morphological alterations are still not known, it is conceivable that collagen III deposition in the anterior vaginal wall affects not only the architecture of the muscle layer but could also modify the intramuscular neurovascularisation and account for an alteration of the neuromuscular plasticity of the layer.

## Introduction

Pelvic organ prolapse (POP) is a pathology that belongs to pelvic floor dysfunctions, characterised by a moving down of the pelvic floor of the vaginal wall and uterus through the urogenital hiatus. Due to the relative positioning between the organs around the pelvis, POP can also be associated with a descent of the bladder, rectum and possibly of the intestinal loops, with different degrees of severity and associations (Haylen et al. 2016). POP is a disease that can have such a highly invalidating impact on a woman's life, in that it totally interferes with physical and mental well‐being and has repercussions for relational, sexual and normal physiological activities (Doaee et al. 2014; Dieter et al. 2015). The incidence of POP is 40% in women aged between 45 and 85 years, and increases with advancing years; in women over 50 years it was estimated that the prevalence is 30–50% and this represents the main clinical indication for hysterectomy (Nygaard et al. 2004; Sliekerten Hove et al. 2009; Von Theobald, 2011). POP is a multifactorial disease, and the main risk factors include pregnancy, delivery, events that induce an increase in intra‐abdominal pressure such as obesity, weight‐lifting, bronchial pneumonia and chronic obstructive pulmonary disease (COPD; MacLennan et al. 2000; Kepenekci et al. 2011; Memon & Handa, 2013; Direkvand‐Moghadam et al. 2014).

Pelvic floor muscles, uterosacral ligaments and endopelvic fascia represent the anatomical support for female pelvic organs, and their histological modifications could play a crucial role in the pathophysiology of the disease (Petros & Swash, 2008; Petros et al. 2008; Kaplan et al. 2011; Vulic et al. 2011).

The vagina is a fibromuscular organ formed by overlapping tunics, including: the mucosa with the epithelium and the lamina propria, the muscularis propria and the adventitia. Type I collagen is made up of large‐diameter fibres; thick and highly resistant to tensile forces, it normally represents the preponderant isotype of the different layers in the normal vaginal wall.

Many recent studies highlight the reorganisation of the muscularis propria architecture of the vaginal wall in the course of POP, observing an increase in type III collagen with very thin and fragile fibres that become unable to support the daily traction that affects the pelvic floor (Alarab et al. 2014). It has been demonstrated that a switch from phenotypic smooth muscle cells (SMCs) to myofibroblast differentiation could be the underlying cause of structural modifications in the tunica muscularis of the anterior vaginal wall. Indeed, SMCs undergo an aberrant switch from a contractile to a synthetic extracellular matrix, producing a phenotype consequent to their trans‐differentiation into myofibroblasts (Smith et al. 1990; Budatha et al. 2013; Severi et al. 2014; Vetuschi et al. 2016). Therefore, the switch from type I to type III occurring in POP entails a greater distensibility, but at the same time fragility, of the wall itself (Tremollieres, 2010; Kannan et al. 2011; Kerkhof et al. 2013). Recent studies (Meijerink et al. 2013; Chen et al. 2015, 2017; Vetuschi et al. 2018) have suggested that advanced glycation end‐products (AGEs) are able to stimulate, through the interaction between AGE and its receptor RAGE, connective remodelling by directly interacting with metalloproteinase 1 (MMP1), tissue inhibitor metalloproteinase (TIMP) and mitogen‐activated protein kinase (MAPK) to promote the physiopathology and progression of POP. By using protein kinases such as extracellular signal‐regulated kinase 1/2 (ERK1/2), AGEs are able to also interact with the transforming growth factor (TGF)‐β/Smads pathway, which is known to affect collagen synthesis and deposition (Meijerink et al. 2013).

Changes in muscularis propria in POP also include a reduction in the concentration of elastic fibres and an imbalance in the extracellular matrix turnover, due to the overexpression of MMPs and a simultaneous reduction in their inhibitors (Budatha et al. 2013; Shynlova et al. 2013; Yucel et al. 2013; De Landsheere et al. 2014; Vetuschi et al. 2018).

Whereas several lines of evidence suggest crucial morphological alterations of muscularis propria in POP, only a few studies investigated the architecture of the neurovascular network in the anterior vaginal wall of women with POP. Perivascular areas of vaginal wall partially lose neuropeptide Y (Hu et al. 2012) and vasoactive intestinal polypeptide, causing impaired muscle functions in women with POP (Busacchi et al. 1999). In addition, abnormalities in general neuronal markers such as neuron‐specific enolase and glial markers (protein‐S100) involved in sensory afferent neural pathways and in sympathetic efferent nerves have been shown in the pelvic floor of women with genito‐urinary prolapse (Busacchi et al. 1999). The contractile function of muscularis propria in both the gut and genito‐urinary tracts is regulated by the interstitial cells of Cajal (ICCs). These c‐kit immunopositive cells are located within the longitudinal and circular muscle layers of the tunica muscularis of the large intestine and within the smooth muscle layer of the vaginal wall, playing a role as pacemakers in the initiation and propagation of electric waves (Vetuschi et al. 2006; Shafik et al. 2007). Numerous studies have reported that a loss or damage to ICC networks is observed in a variety of intestinal motility disorders (Sanders et al. 2002; Ward et al. 2002; Vetuschi et al. 2016), but ICC morphology and their role was not investigated in POP.

Because in POP the vaginal wall exhibits a disorganisation of the muscularis propria, with an increase in type III collagen, MMPs and TIMP due to an involvement of the pathways AGE/ERK1‐2 and TGF‐β/Smads, the aim of the present study was to verify the hypothesis that this connective tissue remodelling could be associated with neurovascular alterations.

## Patients and methods

In this study we examined a total of 40 patients with a clinical history of at least one vaginal delivery. This included 10 control women, without symptoms of urinary incontinence or uterine prolapse, who underwent laparotomic hysterectomy for uterine fibromatosis or other benign gynaecological disorders (control group), and 30 clinical cases from women suffering from at least stage III POP, who underwent colpohysterectomy followed by anterior or posterior plastic surgery. Both controls and patients did not show differences regarding age (average age 57 SD ± 14 years) or body mass index (between 25 and 28 kg m^−2^).

The Institutional Ethic Committee (n. 36944) approved the investigation protocol, and all eligible patients signed a consent form for the processing of personal data and allowing the excision of tissue and its use for this study.

Information related to clinical histories was obtained. None of the subjects included in our study had history of the following: vulvar, vaginal or cervical neoplasia; connective tissue diseases; dystrophic, vulvar or vaginal lesions; hormone‐replacement therapy; incontinence; COPD; constipation or neurodegenerative diseases.

Pelvic organ prolapse biopsies were collected during vaginal plastic surgery, while control fragments were provided during abdominal hysterectomy.

### Histology and immunohistochemistry analyses

All specimens of anterior vaginal wall were obtained during surgery, then cleaned and immediately placed in 4% buffered formalin for 3 h at room temperature. Fragments were embedded in low‐temperature‐fusion paraffin, and paraffin blocks were cut at a thickness of 3 μm for several histological stainings.

Each section was stained with Haematoxylin and Eosin (H&E) in order to evaluate the general architecture of the anterior vaginal wall; the Masson Trichrome stain was used to identify collagen deposition.

For IHC processing, tissue sections were incubated in methanol and 3% hydrogen peroxidase solution for 40 min, and then rinsed in phosphate‐buffered saline (PBS). Thereafter, fragments were incubated overnight at 4 °C with polyclonal antibodies S‐100 (sc‐53438), c‐kit (sc‐168), α‐smooth muscle actin (SMA; sc‐32251; Santa Cruz Biotechnology, Santa Cruz, CA, USA), neurofilament (N4142) and glial fibrillary acidic protein (GFAP; G3893; Sigma‐Aldrich, Saint Louis, MI, USA). All antibodies were diluted 1 : 400. Samples were then rinsed with PBS for 10 min and incubated with a labelled streptavidin‐biotin‐peroxidase conjugate kit (Dako Envision HRP: K 5007, Dako A/S Produktionsvej Glostrup, Denmark) for 20 min. After rinsing in PBS for 10 min, the sections were incubated with 3,3‐diaminobenzidine‐tetrahydrochloride (DAB: K3468, Dako Cytomation, North America, CA, USA) for 1–3 min. Lastly, the samples were counterstained with Mayer's Haematoxylin and observed under a photomicroscope Olympus BX51 Light Microscope (Olympus, Optical, Tokyo, Japan).

To demonstrate the immunoreaction specificity, negative and positive controls were performed for all immunoreactions. As negative controls, sections were treated in an identical manner but with 0.01 m PBS replacing the primary antibody.

All sections were evaluated independently by two experienced pathologists and examined under an Olympus BX51 Light Microscope (Olympus Optical). Quantitative comparison of immunohistochemical (IHC) staining was measured by ImageJ, a digital image analysis public domain software (W.S., Rasband, Image J, US National Institutes of Health, Bethesda, MD, USA; imagej.nih.gov/ij/) for S‐100, GFAP and neurofilament antibodies tested in the study. Five microscopic fields were selected from control and POP groups, photographed at the same magnification and then subjected to IHC profiler (S‐100, GFAP and neurofilament) and ImageJ cell counter plugin (α‐SMA) software analysis. The immunopositivity was expressed as a percentage of the total software‐classified areas, and the data obtained were plotted as histograms. Results were expressed as means ± SD; a *P*‐value ≤ 0.05 was considered statistically significant.

### *In vitro* analyses

Samples collected from patients were stored at −80 °C, and were successively processed for the whole protein lysate extraction and analysis of expression of c‐kit (sc‐168), NF (N4142), S‐100 (sc‐53438) markers (dilution as data sheet suggestion) with a Western blot assay. In particular, the protein lysate was run through a sodium dodecyl sulphate–polyacrylamide gel electrophoresis in denaturant conditions. After that, proteins were transferred to a nitrocellulose membrane and blotted for the specific antibodies of interest in accordance with the protocol mentioned in our previous work (Vetuschi et al. 2018).

### Statistical analyses

Statistical analyses were performed using a Student's *t*‐test between two groups. Results were expressed as means ± SD. A *P*‐value ≤ 0.05 was considered statistically significant.

## Results

### Whole‐mount histology and immunohistochemistry

**Figure 1:**
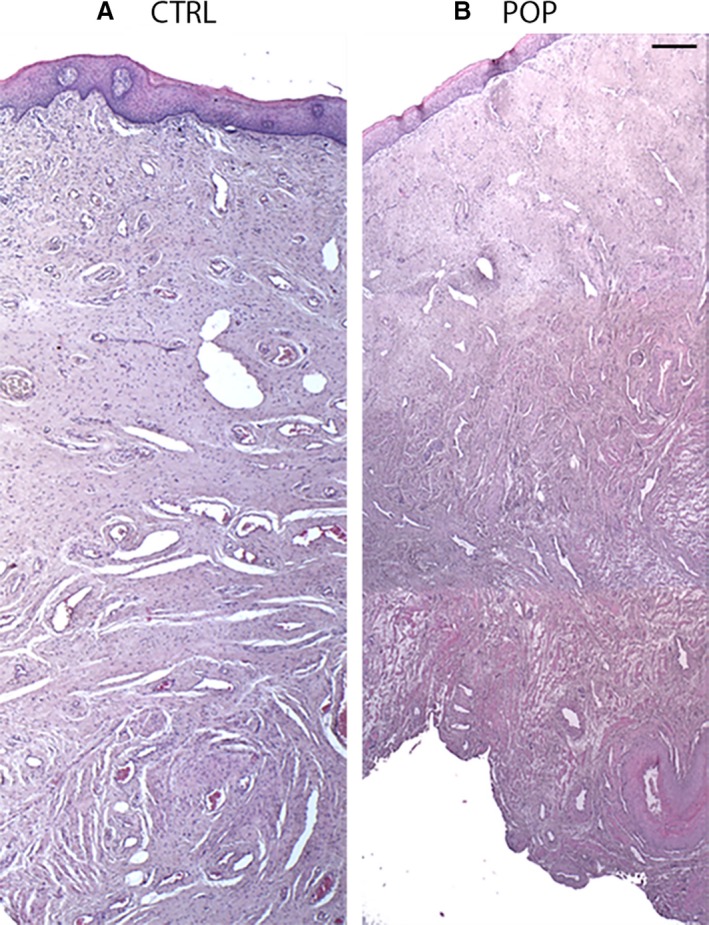
Haematoxylin and Eosin (H&E) staining. Histomorphological features of anterior vaginal wall. Histological analyses highlighted that all layers were present both in (A) control (CTRL) and (B) pelvic organ prolapse (POP) samples. No inflammatory infiltration was detected (A,B). The figures were the result of montage/composite images of three consecutive fields captured at 5 ×. Original magnification (O.M.) 5 ×, Scale bar: 200 μm.

Haematoxylin and Eosin histomorphological analysis showed all the layers constituting the anterior vaginal wall, and the absence of inflammatory infiltration in the subepithelium and lamina propria both in controls (Fig. 1A) and POP samples (Fig. 1B). As previously described (Vetuschi et al. 2016), the Masson Trichromic stain revealed, in the prolapsed specimens, a disruption of SMCs in muscularis propria (data not shown); they were surrounded by an increased amount of collagen fibres resulting in a complete disarrangement of the muscularis layer. This differed from control samples that were arranged into orientated and regular fascicles.

**Figure 2:**
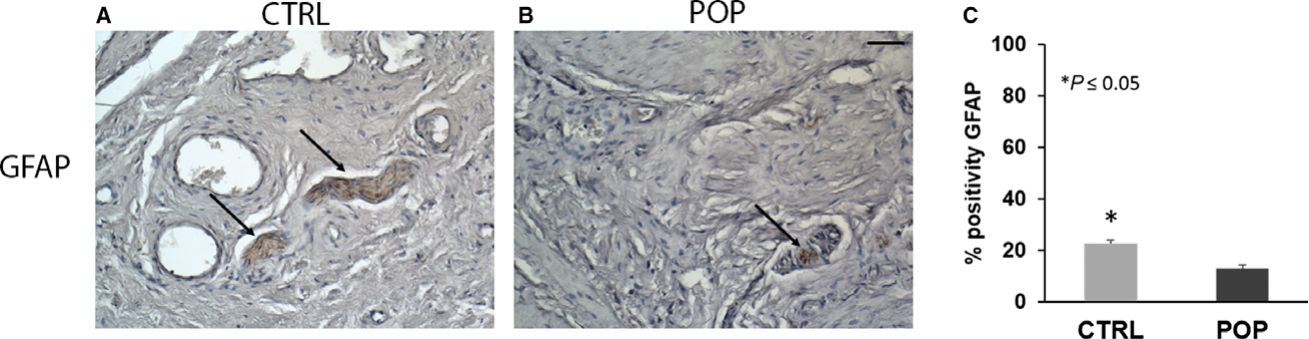
Immunohistochemistry (IHC) for glial fibrillary acidic protein (GFAP). In muscularis propria of pelvic organ prolapse (POP) samples (B), GFAP expression was lower within the supporting cells of small and rare ganglia as compared with specimens from control patients (A). Quantitative data analyses (C) confirmed the IHC results. O.M. 20 ×. Scale bar: 50 μm.

Immunohistochemistry was used to detect the general architecture of the neural network of the muscularis propria in the anterior vaginal wall both in control and in POP samples. GFAP labelling of class III intermediate neurofilaments is expressed in enteric glial cells as part of the enteric nervous system. In the central nervous system, GFAP is located in mature astrocytes and in neural stem cells. GFAP‐β is also highly expressed in non‐myelinated immature Schwann cells in the peripheral nervous system. The GFAP immunoreactivity of the muscularis propria of POP samples showed a lower GFAP expression within the supporting cells of small and rare ganglia (Fig. 2B,C). In controls, we found that GFAP‐bearing glial cells were more numerous as expected in the muscularis tunica, with a normal morphological architecture (Fig. 2A,C).

**Figure 3:**
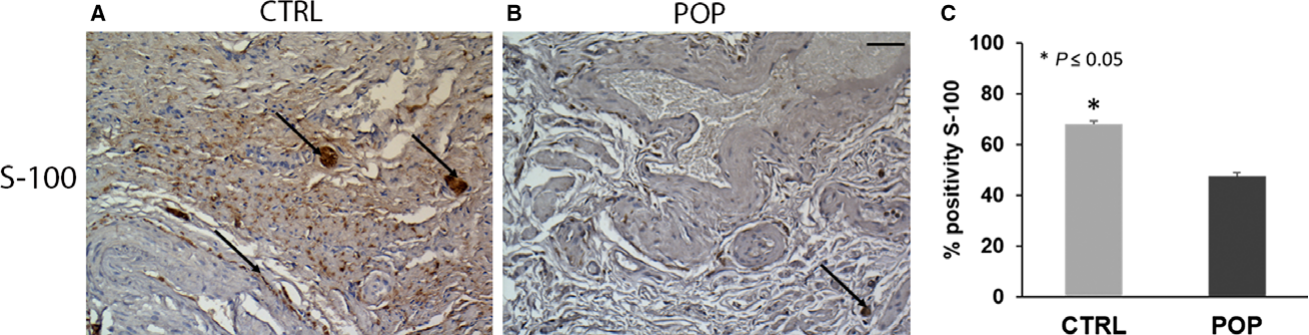
Immunohistochemistry (IHC) for S‐100. The positivity detected for S‐100 was considerably different between the two groups. In control samples (A), large ganglia and nerve bundles were positive within the muscularis; whereas in (B) pelvic organ prolapse (POP), immunopositivity detected small nerve bundles or reticular fibres immersed in the connective tissue. Quantitative data analyses (C) confirmed the IHC results. O.M. 20 ×. Scale bar: 50 μm.

The neuronal network within the muscularis layer was analysed using S‐100, a pan‐neuronal marker that recognised glial/Schwann and astrocyte cells rather than neurons. In samples taken from POP patients, S‐100 immunopositivity was generally decreased with respect to control specimens as confirmed by quantitative analyses (Fig. 3C), and showed small nerve bundles or reticular fibres distributed throughout the connective tissue and interspersed between muscular fibres. In POP, small ganglia were also observed (Fig. 3B) instead of large ganglia, but large nerve bundles were visible in control anterior vaginal samples (Fig. 3A).

**Figure 4:**
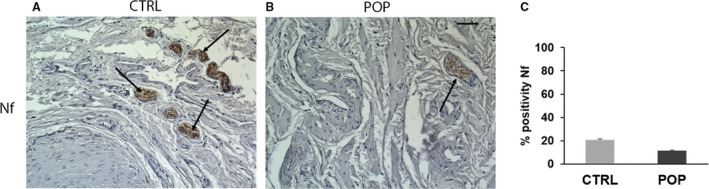
Immunohistochemistry (IHC) for neurofilament. The immunohistochemical evaluation of muscularis propria of (A) controls demonstrated a normal distribution of neurons within the large ganglia as compared with (B) pelvic organ prolapse (POP) samples, in which immunoreactivity was limited to smaller and fewer ganglia. O.M. 20 ×. Scale bar: 50 μm.

**Figure 5:**
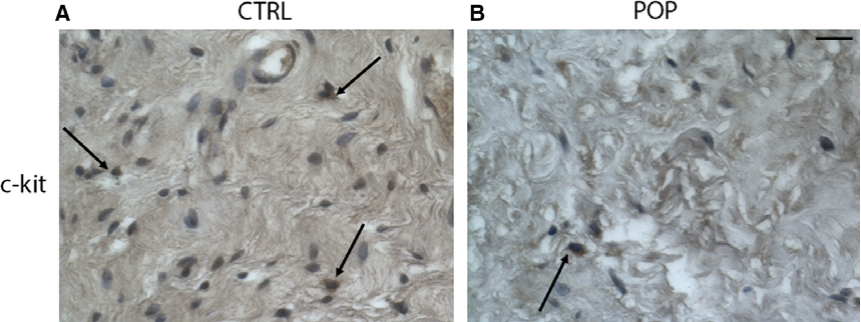
Immunohistochemistry (IHC) for c‐kit. The morphological aspect of interstitial cells of Cajal (ICC) in (B) pelvic organ prolapse (POP) samples was similar to that observed in (A) controls, but they were fewer in number. O.M. 20 ×. Scale bar: 50 μm.

**Figure 6:**
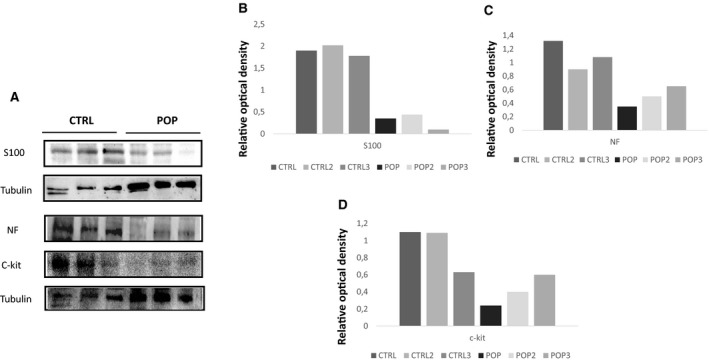
Western blot evaluation of S‐100, NF and c‐kit markers in (A) control (CTRL) and pelvic organ prolapse (POP) samples, and (B–D) relative densitometry quantisation.

The number and dimension of ganglia located in the muscularis layer were evaluated using neurofilament, a marker for labelling type IV intermediate neurofilaments, the expression of which reflects neuronal maturation in both the central and peripheral nervous systems. The IHC evaluation of the muscularis propria of controls demonstrated a normal distribution of neurons within the large ganglia (Fig. 4A); however, in POP samples immunoreactivity was limited to smaller and fewer ganglia (Fig. 4B,C).

c‐kit is a specific marker for ICC that form a network widely distributed within the muscularis propria where they act as pacemaker cells. They are responsible for initiating and modulating electrical waves, and regulate the contractile activity of the tunica muscularis of the vaginal wall as in the other tracts of the genito‐urinary apparatus (Shafik et al. 2005). In control samples, ICC‐positive cells for c‐kit were identified in the muscularis propria (along the neurovascular septa) in between smooth muscle bundles where they appeared separately or in groups. These cells were oblong shape and different sizes, with large and oval nuclei and wavy processes resembling the phenotype of the muscular ICCs within the muscularis gastrointestinal tract, showing immunoreaction for c‐kit (Fig. 5A). In POP samples, the morphological aspect of ICC was similar to that observed in controls but lower in number (Fig. 5B). These IHC data agreed with Western blot analyses performed on representative tissue‐extracted samples for the S‐100, NF and c‐kit markers (Fig. 6A–D).

**Figure 7:**
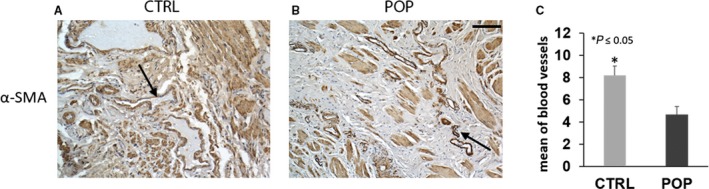
Immunohistochemistry (IHC) for α‐smooth muscle actin (SMA). Significant differences were noted in the number of microvessels and in their thickness between the two groups: (A) in control (CTRL) samples, abundant small vessels and capillaries were observed; whereas (B) in pelvic organ prolapse (POP) samples they were fewer and smaller. O.M. 20 ×. Scale bar: 50 μm.

In order to study the microvascular assessment of muscularis layer, we performed IHC for α‐SMA, a specific marker of SMCs located in tunica media of vessels. In normal samples, abundant small vessels and capillaries were observed and their wall was thinning (Fig. 7A); on the contrary, significant differences were noted in the number of microvessels in POP samples in which the disorganised muscularis appeared characterised by few and small vessels (Fig. 7B,C).

## Discussion

A hallmark of POP is a disorganisation of the muscularis propria, which is increased in thickness and is characterised by a disorganisation of the normal architecture. Muscular fibres appeared disrupted and surrounded by abundant type III collagen that seems to be derived from a phenotype switch of SMCs to myofibroblasts, making it able to produce an extracellular matrix that eventually compromises the dynamic function of the pelvic floor (De Landsheere et al. 2014; Vetuschi et al. 2016, 2018).

It has been demonstrated that in the muscularis propria of POP patients, AGEs are overexpressed and their increase is inversely related to collagen I content, depending on ERK1/2 and Smads pathways activation without involving TGF‐β signalling (Vetuschi et al. 2016, 2018).

Despite several studies discussing the connective and muscular tissue modifications, related to a trans‐differentiation from a contractile phenotype to a synthetic type, there is a paucity of literature evaluating the innervation and microvascularisation pattern related to prolapse status in the human anterior vaginal wall in POP. In the present study, we hypothesised that the decreased content of SMCs and the increased amount of type III collagen are associated with a disruption in innervation and changes in microcirculation. We evaluated the possible contribution of changes in the neuronal network, the number of ICCs and in microvessel density to POP through the comparative IHC analyses of major neurovascular markers.

Our results showed that S‐100 and GFAP had a lower expression in ganglia and nerve bundles of muscularis propria of POP patients vs. controls, in which an intensive network of nerve fibres and large immunopositive ganglia for these antibodies were evident. Concerning NF and c‐kit, similar morphological findings were found in the same layers. In particular, in POP samples both neuronal cells located in ganglia and ICC interspersed within the SMCs were fewer in number, showing an evident depletion differing from the regular distribution and density of the same cells in the tunica muscularis of control specimens.

All these morphological changes, which result in abnormalities of neuronal density, number of ganglia and density of ICCs, disrupt the normal anatomy of the muscularis propria of the anterior vaginal layer and could aggravate POP damage. We observed an IHC decrement of neurovascular markers in POP samples, with a loss of immunoreaction within nerve bundles, ganglia and ICCs. This may cause a failure of initiation, transmission and propagation of muscular contractility, resulting in functional motility and sensory vaginal disorders.

The cellular processes responsible for these neurovascular changes in muscularis propria of POP are still not known. We may hypothesise that collagen type III production and deposition promote the disarrangement of SMCs, and these structural alterations may influence a concomitant ganglioneuritis and a reduction of ICCs that leads to the sequential development of POP.

It is conceivable that in the prolapsed anterior vaginal wall, neuronal and ICCs loss may be related to changes in contractility and tonus of the muscular layer. The denervation and the formation of wide areas of collagen type III deposition within the muscularis propria can modify not only the architecture of the muscle layer but can also alter the intramuscular nerve fibres. One can speculate that all together, these relevant histological changes could affect the neuromuscular plasticity of the anterior vaginal wall and offer a possible explanation for pathogenetic hypothesis in POP.

In conclusion, this preliminary IHC and molecular analysis showed a decreasing of distribution of nerves and microvascular density in the human anterior vaginal wall in POP that could compromise vaginal function. We assume that the extracellular matrix deposition acts in modifying the normal architecture of muscularis propria, together with a rearrangement of the neurovascular network of the same layer, and could account for a causative role in pelvic floor disorders.

These preliminary morphological data could be useful to translate scientific developments into a more effective therapeutic treatment, which involves a combination of neurotrophic, neuroprotective and vasoprotective molecules to surgical approaches. Nevertheless, further investigation is required to confirm the impact of the muscular, neural and vascular alterations we found in the pathogenesis of pelvic floor dysfunctions.

## Conflict of interest

The author(s) declared no conflicts of interest with respect to the research, authorship and/or publication of this article.

## Funding

The author(s) disclosed receipt of the following financial support for the research, authorship and/or publication of this article: This study was supported by grant RIA (Rilevante Interesse di Ateneo) from the University of L'Aquila, Department of Biotechnological and Applied Clinical Sciences, L'Aquila, Italy.

## Author contributions

All authors have contributed to this article as follows: RS and SP developed the study design, coordination and manuscript drafting; SP and GS performed IHC and quantitative analyses; AC and CF were responsible for *in vitro* analyses; GC and ADA provided surgical specimens; AV and EG provided study supervision, as well as critical manuscript revision; and all authors have read and approved the manuscript as submitted.
